# Refining the Role of ERK Signaling in Anthracycline-Induced Cardiotoxicity

**DOI:** 10.1016/j.jaccao.2025.10.005

**Published:** 2026-04-21

**Authors:** Zheyu Wang, Defeng Zhao

We read with great interest the report by Moossavi et al^1^ on the inhibition, but not depletion, of extracellular signal–regulated kinase (ERK) signaling in anthracycline-induced cardiotoxicity. This work provides important insight into the dual role of ERK signaling in cardiomyocyte injury and protection. However, we believe that 2 critical aspects deserve further consideration.

The first concerns the observation that homozygous erk1^−/−^ mutants develop intrinsic cardiac dysfunction and aggravate anthracycline toxicity despite reduced senescence. This finding implies that basal ERK activity is indispensable for short-term cardiomyocyte survival. The need for basal ERK signaling appears to outweigh the potential benefit of attenuating long-term cellular aging. Basal ERK signaling is essential for maintaining myocardial homeostasis and preventing apoptosis in mammalian systems.^2^ Reduced ERK activity is linked to mitochondrial injury, a central feature of anthracycline toxicity. Doxorubicin suppresses mitofusin 2 expression, promotes mitochondrial fission, and increases reactive oxygen species generation, leading to cell death.^3^ Complete ERK depletion may amplify this process by removing its protective influence on mitochondrial integrity, resulting in acute cellular loss distinct from the gradual decline associated with senescence. Future studies should extend beyond senescence markers to quantify indexes of acute cell death and evaluate ERK regulation of mitochondrial proteins in nonregenerative cardiac models.

The second issue relates to the therapeutic window of partial ERK inhibition. Its success depends on suppressing pathologic hyperactivation while maintaining basal phosphorylation necessary for homeostasis. The antiaging role of the ETS factor Aop in *Drosophila* provides a useful framework for understanding this effect. In mammalian cardiomyocytes, excessive ERK activation stimulates the homologous ETS2 factor and nuclear factor of activated T cells (NFAT), driving transcription of prohypertrophic genes, including ANP, BNP, and microRNA-223.^4^ Under anthracycline stress, ERK activity may reach a level sufficient to trigger this maladaptive ETS2 and NFAT pathway, promoting hypertrophy and senescence. Partial inhibition may protect the heart by selectively restraining this high-affinity pathologic arm while preserving basal phosphorylation that supports cell survival. This fine regulation is likely mediated by cardiac mitogen-activated protein kinase scaffolds that spatially organize ERK signaling and determine which downstream effectors are activated at distinct concentration thresholds.^5^ Future research should define the quantitative level of phosphorylated ERK required for pathologic remodeling vs the threshold sustaining mitochondrial stability and survival.
